# Advancing Tobacco Control Through Point of Sale Policies, Providence, Rhode Island

**DOI:** 10.5888/pcd16.180614

**Published:** 2019-09-19

**Authors:** Deborah N. Pearlman, Jasmine A. Arnold, Geri A. Guardino, Erin Boles Welsh

**Affiliations:** 1 Brown University School of Public Health, Providence, Rhode Island; 2 Rhode Island Department of Health, Providence, Rhode Island

## Abstract

Local point of sale (POS) policies are key strategies for preventing and decreasing tobacco use among youth. In January 2013, Providence, Rhode Island implemented a comprehensive POS tobacco policy restricting the sale of flavored tobacco products and discounts of tobacco product prices. Lack of sustained funding for enforcement has been challenging. Our research focuses on the policy evaluation after enforcement began. We observed a decrease in availability of flavored tobacco products as citations for violations increased. However, we observed little change in the availability of flavored tobacco products with ambiguous descriptors that connote a flavor. Current use (within 30 days before survey) of tobacco products among high school students declined after the policy was enforced. Collectively, these findings demonstrate that POS tobacco policies are effective. The tobacco industry’s marketing of products that do not explicitly reference flavors might undermine enforcement of POS tobacco restrictions in Providence and elsewhere in the United States.

**Summary**

**What is already known about this topic?**

Evaluation of point of sale (POS) policies that restrict the sale of flavored tobacco products is a new area of research and evidence of its effectiveness is limited.

**What is added by this report?**

Rigorous enforcement of Providence, Rhode Island’s flavored tobacco restrictions and price discounting resulted in increased citations of policy violations over 2 years. High school students’ current e-cigarette use decreased by 7 percentage points from preenforcement to postenforcement.

**What are the implications for public health practice?**

Findings from this study highlight the need for new approaches to POS tobacco policy evaluations. Such policies might be undermined by the tobacco industry’s increased marketing of products with ambiguous flavor descriptions.

## Introduction

The retail environment or point(s) of sale (POS) is currently the primary venue where tobacco companies market their products in the United States. In 2016, the US market had 565 unique e-cigarette brands (1), many marketed in distinct flavors (2), and more than 250 unique cigar flavors (3). Flavored tobacco products are heavily marketed in convenience stores, places that adolescents visit at least once per week (4). E-cigarettes are the most commonly used tobacco product among adolescents (20.8%), followed by cigarettes (8.1%) and cigars (7.6%) (5). The popularity of e-cigarettes is attributed, in part, to the sale of these products in flavors that appeal to youth (1). Of equal concern is the proliferation of tobacco products with text or images that indicate a flavor without specifically naming the flavor (6), hereafter referred to as “not clearly labeled.” Studies provide evidence that frequent exposure to retail tobacco marketing encourages youth smoking (7–9). Evaluations of local POS tobacco control policies to prevent and reduce youth smoking are still at early stages.

## Purpose and Objectives

In January 2012, the city of Providence passed a local POS tobacco policy to restrict the sale of flavored tobacco products and limit price promotions that make tobacco products cheaper and more accessible. In doing so, Providence, Rhode Island, became the first city in the United States to restrict tobacco price discounting and multipack offers and the second city to limit the sale of flavored tobacco products (excluding menthol), except in legally permitted tobacco bars. Providence requires city tobacco retailers to apply annually for a license, with escalating penalties for policy violations up to license revocation. Penalties assessed for violations provide a funding stream for enforcement. Originally slated to take effect on March 1, 2012, the policy was challenged in the courts by the tobacco industry, but the court ruled in favor of the city (10). The 3 policy strategies were implemented in January 2013 and became the Rhode Island Model Tobacco Policy. By 2017, Providence had a comprehensive retail POS tobacco policy for 5 years. Enforcement of the policy by Providence police was complex and challenging. First, local resources were not enough to sustain compliance check inspections of tobacco retailers. Second, enforcement officers did not have compliance check inspection forms tailored to the city’s ordinances, which were needed to improve data collection.

We evaluated the effects of Providence’s POS tobacco policy after the Rhode Island Department of Health (RIDOH) Tobacco Control Program was awarded a 2-year Centers for Disease Control and Prevention (CDC) grant in 2017, which supported rigorous enforcement of the policy. Our aims were to

1. Determine whether both flavored and nonflavored tobacco products and tobacco price promotions are readily available at retail POS.
2. Determine whether citations for illegal sale of flavored tobacco products and tobacco price promotions increased with enforcement and then declined, as retailers were educated about the model policy.
3. Examine whether enforcement of the POS tobacco policy decreased youth smoking.

## Intervention Approach

In 2015, the RIDOH Tobacco Control Program was one of 5 states to be awarded a 2-year CDC competitive grant. The grant supported the community infrastructure needed to advance the adoption of the Rhode Island Model Tobacco Policy in 6 cities statewide, as was successfully done in the city of Providence. The Providence Healthy Communities Office authored a Tobacco Point of Sale Enforcement Toolkit. The city’s enforcement unit provided technical assistance to other grantees on enforcement of local POS tobacco policies. The 2-year grant provided a strong foundation for an additional 2 years of CDC funding to support the implementation and evaluation of the Rhode Island Model Tobacco Policy in the towns of Barrington, Johnston, and West Warwick and the cities of Central Falls, Providence, and Woonsocket. Although not funded, the RIDOH Tobacco Control Program partnered with the town of Middletown to support implementation of the Rhode Island Model Tobacco Policy.

With new grant funding, the Providence Healthy Communities Office conducted observational retail store assessments using the national Standardized Tobacco Assessment for Retail Settings (STARS) (11) adapted for Rhode Island (RI-STARS). RI-STARS is a paper-and-pencil form designed to assess the availability, placement, and pricing of flavored tobacco products that are clearly labeled and those not clearly labeled at retail POS. Retailer education was included at the end of each visit to ensure vendors complied with Providence’s tobacco ordinances. Providence law enforcement officers were responsible for conducting compliance checks with forms tailored to the city’s policy and penalty structure. Actions requiring enforcement were from 3 separate RIDOH compliance check forms: 1) sales of tobacco products to minors, 2) sales of flavored tobacco products to underaged youth and adults, and 3) price discounting and multipack offers. A tobacco retail violation form documents violations and adjudication actions through Providence’s Board of Licensing and District Court. RI-STARS and RIDOH compliance checks use Garcia y Vega Game Blue cigarillos (Game Blue) as a measure of the availability of a known flavored product that is not clearly labeled as such. Game Blue is a product used specifically for comparison on enforcement and RI-STARS forms.

## Evaluation Methods

Two rounds of store observation audits with retailer education and 5 rounds of RIDOH retail compliance checks were conducted during the study period. Stores for on-site observations and the first round of compliance checks were randomly selected from the Rhode Island Taxation List of Providence tobacco retailers (n = 445). In the 4 subsequent rounds of compliance checks, stores found to be in violation of the Rhode Island Model Tobacco Policy were kept in the sampling frame. Additional stores were then randomly selected from the taxation list so that each round of compliance checks had between 65 and 200 stores for enforcement, depending on the availability of funding. US Food and Drug Administration (FDA) compliance inspections were collected every 6 months to identify stores cited for violations. Stores found to be in violation of federal tobacco laws were cross-checked with RIDOH compliance check forms to identify repeat offenders who were illegally selling tobacco products to minors.

Data on adolescents’ current use of tobacco products were obtained from the 2012, 2016, and 2018 Annie E. Casey Evidence2Success Providence Youth Experience Survey (YES) (12). In 2012, Providence, Rhode Island, became the first site to adopt the Annie E. Casey Foundation’s Evidence2Success framework and implement the YES. YES is a cross-sectional, self-administered, anonymous, school-based survey that tracks trends in child well-being. The Providence high school YES was implemented as a census survey to collect information in classrooms from all 10th and 12th grade students at the time of administration. Current use (within 30 days before survey) of cigarettes was measured in all 3 survey years. Questions about other tobacco use were asked in 2016 and 2018. All tobacco questions were coded as binary (0 or 1) variables. Students who said they did not answer the surveys honestly were excluded from analyses. The final analytic samples in the 2012, 2016, and 2018 YES were 2,150, 2,062, and 2,223, respectively.

Overall differences across years were assessed by using 1-way analysis of variance (ANOVA), by α of 0.05, and by overlapping 95% confidence intervals (CIs). Data were analyzed by using SAS 9.4 (SAS Institute, Inc).

## Results

Aim 1 was to determine whether both flavored and nonflavored tobacco products and tobacco price promotions were readily available at retail POS. RI-STARS store audits were completed in 90 stores in October 2017 and 82 stores in January 2018 (Table 1). Analysis of observational data showed that the availability of flavored products decreased from 37 of 90 stores in Round 1 (41%) to 14 of 82 stores in Round 2 (17%), a decrease of 24 percentage points. The availability of clearly labeled cigarillos and cigars also decreased at retail POS. Game Blue cigarillos remained accessible in 76% of store audits in Round 1 and 73% of store audits in Round 2. Approximately half of the stores visited sold discounted cigarettes. None of the 55 stores visited in December 2018 were observed to have Game Blue cigarillos (the 4th and final round of store audits). Clearly labeled flavored cigarillos, premium large cigars, and e-juices were observed in 2% of stores. Coupon cigarette price promotions were observed in 11% of stores visited.

**Table 1:** RI-STARS Tobacco Product Observed Availability, Providence, Rhode Island, 2017 and 2018

RI-STARS Observed Availability	Round 1: October 2017 (N = 90 Stores)	Round 2: January 2018 (N = 82 Stores)
**Stores with clearly labeled flavored products**	37 (41%)	14 (17%)
**Products observed**		
Cigarillos	30 (33%)	5 (6%)
Cigars	21 (23%)	2 (2%)
Smokeless tobacco^a^	8 (9%)	1 (1%)
E-cigarettes	2 (2%)	1 (1%)
E-liquids	19 (21%)	12 (15%)
**Stores with not clearly labeled flavored products**	68 (76%)	60 (73%)
**Stores with price promotions**	40 (44%)	40 (49%)
Buy-one-get-one	13 (14%)	5 (6%)
Coupons	40 (44%)	37 (45%)

Abbreviation: RI-STARS, Rhode Island State Tobacco Assessment for Retail Settings.

a Smokeless tobacco included chew, snuff, dip, and snus.

Aim 2 was to determine whether citations for the sale of flavored tobacco products and tobacco price promotions increased with enforcement and then declined as retailers were educated about the model policy. During 9 months, there were 110 RIDOH compliance check inspections of tobacco retailers for sales of tobacco to a minor, 378 RIDOH compliance checks for sales of flavored tobacco products, and 15 RIDOH compliance checks for price discounting of cigarettes. Most stores were found to be compliant with Providence’s POS tobacco policies (n = 413; 82%). The 91 stores cited for a violation had repeated (up to 4) compliance checks. Between the first and last rounds of compliance checks, violations for sale of tobacco to a minor decreased by 12 percentage points to 2%; flavored tobacco adult sale violations (clearly and not clearly labeled products) increased by 20 percentage points to 22%; and violations for price discounting increased by 10 percentage points to 70% (Table 2). Compliance check inspections continued through the end of the 24-month grant. This resulted in an additional 55 compliance checks for tobacco sales involving minors, 127 compliance checks for sales of flavored tobacco, such as, e-cigarettes, cigars, and hookah tobacco, and 32 compliance checks for cigarette price discounting.

**Table 2:** Compliance Checks for Tobacco Points of Sale, Providence, Rhode Island, 2017 and 2018^a^

Compliance	RIDOH Compliance Checks	
	Round 1: November 2017 (N = 99 Checks)	Rounds 2–5: February–July 2018 (N = 408 Checks)
**Youth tobacco compliance checks**	50 (56%)	61 (15%)
**Youth tobacco sale violations**	7 (14%)	1 (2%)
Clearly labeled flavored products	2 (29%)	0
Not clearly labeled flavored products^b^	4 (57%)	1 (100%)
Not flavored cigarillos	1 (14%)	0
**Flavored tobacco product compliance checks**	44 (49%)	334 (82%)
**Flavored tobacco adult sale violations**	1 (2%)	72 (22%)
Clearly labeled flavored products	0	37 (51%)
Not clearly labeled flavored products^b^	1 (100%)	35 (49%)
**Price discounting compliance checks**	5 (6%)	10 (2%)
**Price discounting adult sale violations** c	3 (60%)	7 (70%)

Abbreviation: RIDOH, Rhode Island Department of Health.

a Providence, Rhode Island, compliance check inspections of tobacco retailers during 9 months (November 2017–July 2018).

b Garcia y Vega Game Blue Cigarillos, a product used specifically for comparison in this survey.

c Price discounting violations were for non-flavored conventional cigarettes.

By the end of the 2-year grant, 9 stores were cited for youth violations. Seven stores received a warning, 1 store received a fine of $250. At the time of this report, the case against 1 store was pending. Of the 85 stores cited for flavor sale violations, 72 cases were adjudicated. Two stores were given warnings, 1 store received a fine of $100, 52 stores received fines of $250, 11 stores were fined $350, 1 store was fined $400, and 5 stores received fines of $500. Cases brought against 9 of the remaining 13 cases were dismissed. Three stores closed before their cases were heard in Providence’s Board of Licensing and District Court. Thirteen stores were cited for price discounting violations. Five stores received a fine of $250 and 1 store was fined $600. Seven stores had their cases dismissed. The city of Providence imposes a fine of $250 for the first policy offense, $350 for the second policy offense, and $500 for any subsequent policy offenses. Tobacco retailers with more than 3 offenses are subject to license revocation.

FDA inspectors conducted 496 undercover inspections of Providence tobacco retailers during the 2-year grant period (Table 3). The FDA cited 46 stores for tobacco sales to minors; 20 stores received warning letters and 26 received civil money penalties. Three tobacco retailers were cited for violating Providence’s POS tobacco policy and FDA restrictions.

**Table 3:** Youth Tobacco Compliance Checks, US Food and Drug Administration, Providence, Rhode Island, March 2017–March 2019

Violation	Inspections (N = 496)
**Youth tobacco sale violations**	46 (9.3%)
Warning letter	20 (43.5%)
Civil money penalty	26 (56.5%)

Aim 3 was to examine whether enforcement of the POS tobacco policy decreased youth smoking. The percentage of high school students who reported currently smoking cigarettes was significantly higher in 2016 (7.6%) than in 2012 (3.2%; Table 4). Current cigarette smoking declined by 4.6% after enforcement began in 2016 and was 3.0% in 2018. By contrast, the Rhode Island Youth Risk Behavior Survey (YRBS), which is a representative sample of Rhode Island public high school students, found that the prevalence of current cigarette smoking was 11.4% (95% CI, 9.0%–14.4%) in 2011 and 6.1% (95% CI, 4.3%–8.7%) in 2017.

**Table 4:** Tenth- and Twelfth-Grade Student Use of Tobacco Products, Providence, Rhode Island, 2017 and 2018^a^

Survey Year	Sample Size	% Yes (95% Confidence Interval)				
		Cigarettes	Cigars and Cigarillos	E-cigarettes	Hookahs	Any Tobacco Product^b^
**2012** (POS policy passed January 2012; implemented January 2013)	2,150	3.2 (2.4–4.0)	NA	NA	NA	NA
**2016** (3 years post implementation of policy)	2,062	7.6 (6.3–9.0)	7.1 (5.7–8.5)	13.3 (11.4–15.1)	13.5 (11.6–15.3)	22.2 (20.0–24.3)
**2018** (5 years post implementation of policy)	2,223	3.0 (2.1–3.8)	1.9 (1.2–2.6)	6.6 (5.3–7.8)	7.7 (6.4–9.2)	12.1 (10.5–13.7)

Abbreviations: POS, point of sale; NA, not asked.

a Students were asked, “Which of the following tobacco products have you tried in the past 30 days?” From the Annie E. Casey Foundation Evidence2Success Youth Experience Survey (YES), Providence, Rhode Island.

b Smoked cigarettes, cigars, cigarillos, electronic vapor products, hookah, or used smokeless tobacco or other unspecified tobacco product.

Between 2016 and 2018, current use of any tobacco product declined significantly, from 22.2% to 12.1%. E-cigarettes declined from 13.3% (95% CI, 11.4%–15.1%) to 6.6% (95% CI, 5.3%–7.8%) during 2 years (Table 4). By contrast, the YRBS reported the prevalence of current e-cigarette use among Rhode Island high school students was 19.3% (95% CI, 16.1%–22.8%) in 2015 and 20.1% (95% CI, 16.9%–23.7%) in 2017.

## Implications for Public Health

Providence’s POS tobacco policy represents an important public health achievement. For the first time, the city of Providence had the ability to compare the availability of clearly and not clearly labeled flavored tobacco products at retail POS. The findings from store observations have policy implications. Game Blue cigarillos, a flavored product that is not explicitly labeled as flavored, remained accessible in most stores surveyed through July 2018. By fall 2018, Game Blue was no longer available in stores surveyed (data not shown). On-site retailer education likely contributed to the observed decrease, as did enforcement of Providence’s POS tobacco policy.

Law enforcement officers demonstrated that the newly designed compliance check forms were suitable for monitoring tobacco sales to minors, flavored tobacco sales, and discount restrictions. Rigorous enforcement during 2 years resulted in 107 individual store violations; 79% were for sale of flavored tobacco products. A study of flavored e-cigarette sales as a percentage of all e-cigarette sales in the United States from 2012 to 2016 found that Rhode Island was the only state with a significant decline in flavored e-cigarette sales from 2015 through 2016 (1). Although we cannot attribute these findings solely to Providence’s POS policy, the city’s restriction on the sale of flavored tobacco products might have contributed to this decline. Evaluations of POS tobacco policies that restricted the sale of flavored tobacco products have been conducted in New York City (13,14), Minneapolis and Saint Paul, Minnesota (15), and Massachusetts (16). These policies take different approaches, but the evaluations show that the availability and sale of these products declined significantly after policy enforcement. Minneapolis also saw a significant reduction in the availability of tobacco products with ambiguous flavor names after the flavor restriction was implemented (15). The short-term benefits (within 1 or 2 years after policy implementation) of local-level policy restrictions are promising. Still, enforcement of flavored tobacco bans is difficult. Providence has no mechanism for testing ambiguously labeled flavored tobacco products at a retail POS or when a case is challenged in court. The tobacco industry’s increased marketing of products by concept (“Jazz”) or by characterizing flavors (fruit), rather than using clearly descriptive names, might undermine enforcement of POS tobacco policies in Providence and elsewhere in the United States (6,17).

Providence’s law enforcement officers showed that enforcement of a ban on price discounting, which no other city or town in the United States had yet tried, was possible. Enforcement of this ban is complex. Tobacco products scanned with a price promotion set by the tobacco industry show the reduced price directly on the register without the deduction taken. This presents major challenges to preventing this type of price marketing by the tobacco industry.

One strength of the policy evaluation is that it expanded monitoring of the tobacco landscape in Providence to include FDA compliance inspection data. During the study period, the city of Providence and the FDA conducted separate undercover youth buying inspections to stop illegal tobacco sales to minors. Three stores were cited by FDA and RIDOH for selling tobacco products to an underaged youth. Penalties increased significantly for repeat offenders. FDA and RIDOH enforcement activities likely contributed to the decline in teen use of cigars and cigarillos, e-cigarettes, and hookah tobacco, which are marketed with flavors that appeal to youth. The findings from our analysis of school survey data demonstrate the importance of ongoing enforcement of local ordinances and federal laws to prevent flavored tobacco product sales to underaged youth.

The tobacco research field has an unprecedented opportunity to evaluate POS tobacco policies. Findings from this study are promising. More research is needed to build a strong empirical evidence base that regulating POS tobacco access, availability, and marketing decreases early initiation and continued use of heavily marketed flavored tobacco products among children and youth. Additional research is needed to evaluate POS tobacco policies in the context of other population-based tobacco prevention and control efforts.
